# Universal Single-Cell Transcriptomic Aging Clock powered by LLMs reveals targets to slow cellular aging

**DOI:** 10.1093/geroni/igaf122.4305

**Published:** 2025-12-31

**Authors:** Raghav Sehgal

**Affiliations:** Yale University, New Haven, Connecticut, United States

## Abstract

Large language models such as GPT have shown impressive performance in capturing structure and meaning from natural language. We adapt this capability to biology by representing single-cell transcriptomes as ordered sequences of gene names, analogous to sentences in text. Each cell’s expression profile is converted into a “cell sentence,” enabling pretrained language models to be fine-tuned directly on single-cell data for age prediction. Trained on millions of cells spanning diverse tissues and life stages, the model learns a universal latent representation of aging. It captures both cell-type–specific trajectories and conserved molecular signatures, generalizing across tissues and species. Unlike other foundational models that must be trained from scratch on omics data, our framework leverages already well-validated LLMs, taking advantage of their robust priors while avoiding the cost and instability of building new architectures. The resulting clock achieves state-of-the-art accuracy in predicting biological age at single-cell resolution. Embeddings highlight aging-associated processes such as immune activation, proteostasis decline, and metabolic shifts, aligning with known hallmarks of aging. Importantly, the model can also identify genes whose modulation decreases predicted transcriptional age, nominating candidate targets for therapeutic intervention. These features make the approach valuable both for measuring aging and for prioritizing interventions that may slow or reverse it. This work establishes the first universal, multi-tissue aging clock at single-cell resolution, demonstrating that pretrained language models can be directly adapted to biological data to map, measure, and potentially modify cellular aging.

